# ‘I was a bit hasty … I was a young resident!’ Medical residents' responses to clinical uncertainty

**DOI:** 10.1111/medu.70182

**Published:** 2026-01-21

**Authors:** Nicolas Belhomme, Alain Lescoat, Pierre Pottier, Yoann Launey, Emmanuel Triby, Thierry Pelaccia, François Robin

**Affiliations:** ^1^ Service de Médecine Interne et Immunologie Clinique CHU Rennes Rennes France; ^2^ Université de Rennes, CHU Rennes, Inserm, EHESP, Irset (Institut de Recherche en Santé, Environnement et Travail)—UMR_S 1085 Rennes France; ^3^ Laboratoire Interuniversitaire des Sciences de l'Éducation et de la Communication (LISEC), UR_2310 université de Strasbourg Strasbourg France; ^4^ Service de Médecine Interne et Immunologie Clinique, CHU Nantes, Université de Nantes Nantes France; ^5^ Réanimation Chirurgicale, Département d'Anesthésie‐Réanimation‐Médecine Périopératoire, CHU Rennes Université Rennes Rennes France; ^6^ Service d'Urgences (SAMU 67), CHU de Strasbourg Strasbourg France; ^7^ Centre de Formation et de Recherche en Pédagogie des Sciences de la Santé, Faculté de Médecine université de Strasbourg Strasbourg France; ^8^ Service de Rhumatologie, CHU Rennes Rennes France; ^9^ Université de Rennes, INSERM, Inrae, CHU Rennes, UMR 1317, UMR 1341, Institut NuMeCan (Nutrition, Metabolisms and Cancer) Rennes France

## Abstract

**Introduction:**

Uncertainty is intrinsic to medical practice. Improving trainees' uncertainty tolerance requires exploring their responses to clinical uncertainty in clinical contexts. Although previous research works have highlighted the role of self‐assessment, contextual cues and responsibility, existing models—developed for experienced physicians—often fail to capture residents' intuitive, situated responses. This study explores residents' behavioural responses to clinical uncertainty, focusing on how contextual features shape their actions and decision making. Following Hillen et al., we define behavioural responses as the actions individuals take to cope with uncertain situations.

**Methods:**

Using an interpretative paradigm, we conducted a thematic analysis of semi‐structured interviews with residents from several medical specialties. Considering that age, gender and clinical experience shape responses to uncertainty, we used a maximum variation sampling strategy to ensure diversity in year of residency and gender among participants. Audio‐recorded interviews were conducted following a pretested interview guide focusing on residents' lived experiences of uncertainty and transcribed into verbatims. Analysis combined deductive coding, informed by Hillen's framework and Han's taxonomy, with inductive theme generation to capture novel insights.

**Results:**

Fourteen participants described three main behavioural responses to clinical uncertainty, aligned with Han's taxonomy: reducing uncertainty, protection and adaptation. Their responses were determined by situational determinants, including the patient, the problem at hand, the environment and their individual characteristics. Over time, participants progressed from avoiding uncertainty or relying on supervisors to taking a more systemic and situated approach, integrating a combination of complementary strategies to balance the objectives of patients and physicians. This approach fostered the development of competence in navigating complex clinical situations.

**Discussion:**

Our study shows that uncertainty is a situated experience shaped by dynamic interactions between practitioners and context. Recognising this helps move beyond a purely cognitive view, framing uncertainty as a core competency developed through experiential learning and supported by adaptive strategies.

## Abstract 2

This study reveals that residents learn to live with uncertainty–not eliminate it. Through experience and reflection, they integrate it as a core clinical skill shaped by context and practice. #MedEd

## 1 INTRODUCTION

Uncertainty, defined as the self‐awareness of a lack of knowledge when facing a clinical problem,^1^ is an inherent feature of medical practice. Inadequate management of uncertainty leads to harmful consequences at several levels.^2^ For practitioners, and particularly medical trainees, inadequate uncertainty management is associated with anxiety, loss of confidence, demotivation and burnout.^3^, ^4^, ^5^, ^6^, ^7^ For patients, inadequate uncertainty management weakens the therapeutic relationship, leading to mistrust of the medical profession and dissatisfaction with care.^2^, ^8^, ^9^ At the health care system level, uncertainty contributes to medical errors and the misuse of diagnostic resources.^10^, ^11^ However, growing evidence highlights the constructive role of clinical uncertainty in supporting practitioners' professional development. When clinicians perceive uncertainty as a marker of the limits of their current knowledge, it can stimulate the acquisition of new resources and skills needed to address the situation at hand. Acknowledging one's uncertainty also reflects epistemic maturity: the ability to recognise personal limitations and to adopt a representation of clinical problems that accommodates ambiguity and grey zones, an attitude that resonates with the concept of ‘adaptive expertise’ and with the stance of humility advocated by Han.^12^, ^13^ Considering this dual perspective—uncertainty as both a potential source of negative outcomes and an inherent, productive feature of clinical practice—medical education programmes in North America, the United Kingdom, and more recently continental Europe have increasingly positioned the preparation of learners for clinical uncertainty as an explicit curricular objective.^14^, ^15^, ^16^, ^17^, ^18^

Training residents to tolerate and manage uncertainty requires an understanding of how they experience it, to better identify training needs. Previous studies have shown that residents' experiences are shaped by their performance in dealing with the uncertain situation at hand, as assessed by themselves and their supervisors.^19^, ^20^, ^21^ This self‐assessment determines whether their experience of uncertainty is positive or negative, guides their delayed emotional and cognitive responses and affects their ability to transform their confrontation with uncertainty into transferable learning. Therefore, residents' behavioural responses to uncertainty—defined by Hillen et al. as the actions individuals take to cope with uncertain situations ‐ profoundly shape their overall experience of uncertainty.

In this regard, Stephens et al. described a range of responses to uncertainty among early clinical students, from information seeking to avoidance.^22^ However, these findings are constrained by the limited responsibility and decision‐making authority of such students. As shown in a previous work, the experience of uncertainty is strongly shaped by one's level of engagement in the clinical task, often expressed in terms of assumed responsibility.^19^ Consequently, the way early students experience uncertainty likely differs substantially from that of residents, who face greater autonomy and must sometimes respond independently in urgent situations. The coping resources and behavioural responses observed among junior students therefore appear more limited and may not be directly transferable to residents.

Using the cues utilisation framework, Ilgen et al. showed that trainees struggled with the legitimacy about their appraisal of the problem and their interpretation of the situational cues.^20^ This scepticism prompted safety‐oriented responses, with junior residents managing perceived risks and their lack of confidence by seeking supervision or engaging in cognitive rehearsals and planning strategies. These responses reflected their priority of ensuring safety rather than acting rapidly or independently, and were therefore limited both in diversity and in the range of cues mobilised. Although highlighting links between responses and sources of uncertainty, this work did not establish how specific situational determinants shaped behavioural responses. As such, further insight into how more experienced residents in real practice environments adapt their responses by integrating contextual factors is still needed.

With this in mind, Helou et al. argued that practitioners primarily seek to classify uncertainty as reducible (a lack of information that can be remedied) or irreducible, to determine the most appropriate responses.^23^ Such a process would be grounded in reflection, building on the seminal work of Renée Fox, who distinguishes three types of uncertainty: type 1 (lack of individual knowledge), type 2 (intrinsic limitations of medical knowledge) and type 3 (inability to categorise one's uncertainty).^24^ Helou's model is consistent with Han's taxonomy of strategies, which organises possible responses according to their effects on a continuum ranging from reducing uncertainty to protecting oneself from its adverse effects.^25^ Although these approaches offer a relevant decision‐making model for experienced practitioners, their transferability to the specific population of residents is still to be determined. In a previous study, we demonstrated that residents' responses are primarily determined by the characteristics of the situation and their perceived level of responsibility, rather than by a conscious categorisation of uncertainty.^19^ Similarly, describing the actions taken as ‘strategies’ suggests a conscious, deliberate and anticipatory process. However, numerous studies indicate that, in most cases, decision making is driven by intuitive processes based on experience and pattern recognition rather than by conscious, planned analysis.^26^, ^27^, ^28^ Therefore, although the concept of strategy provides a systematic and comprehensive categorisation of possible responses to uncertainty, it may not accurately reflect how trainees respond to uncertainty in their daily practice. The aim of this study was therefore to describe the range of residents' behavioural responses to clinical uncertainty, as shaped by the dynamic interaction between the individual and the specific characteristics of each situation.

## 2 MATERIALS AND METHODS

We conceptualised uncertainty as a fundamentally subjective and evolving experience rooted in a given context. To guide our analysis, we adopted an interpretative paradigm.^29^ This epistemological stance recognises that the meaning participants give to situations can only be understood through their narratives, which are co‐constructed in interaction with researchers. From this perspective, we conducted a thematic analysis using the approach described by Braun and Clarke.^30^, ^31^ Thematic analysis was particularly suited to our objective as it enabled us to identify themes in participants' narratives reflecting their behavioural responses to uncertainty, by combining deductive analysis using categories from existing literature with an inductive approach open to the emergence of new, data‐specific themes. The research team consequently engaged in a collective reflective process throughout the study. The researchers compared their perspectives at each stage of the research process, questioning how their individual backgrounds and experiences contributed to their interpretations of the data. All members of the research team are involved in clinical care, including on‐call duties, across different specialties. NB, AL and PP are specialists in internal medicine and clinical immunology; FR is a rheumatologist; YL is an intensive care specialist; and TP is an emergency physician. NB is a doctoral student in educational sciences and ET and TP hold PhDs in educational sciences.

### 2.1 Theoretical framework

We used the theoretical framework developed by Hillen et al. to identify themes reflecting participants' behavioural responses to uncertainty.^32^ Based on a systematic analysis of uncertainty measurement instruments, Hillen's framework is widely used in research on uncertainty in medical education. This framework has demonstrated good transferability in previous work including medical students and, in a study conducted by our group, residents, supporting its relevance for our population.^19^, ^22^, ^33^, ^34^ Its structure allows for a holistic examination of learners' experiences of clinical uncertainty by integrating its sources, the spectrum of cognitive, emotional and behavioural responses, and the moderating factors shaping these responses. In addition, Hillen's model offers a comprehensive yet flexible architecture that can be meaningfully combined with Han's taxonomy of uncertainty responses.

For the purposes of this study, we organised Hillen's behavioural response dimension according to their intended effect on uncertainty within Han's framework, thereby enhancing conceptual clarity and enabling a more nuanced analysis of how residents navigate, reduce or tolerate uncertainty in everyday clinical practice.^25^ We therefore employed the primary themes of this taxonomy—uncertainty reduction, adaptation and protection—as a sensitising concept for our analysis of behavioural responses. However, because Hillen's framework was developed using non‐contextualised measurement instruments, it does not explicitly examine how behavioural responses relate to situational factors specific to clinical contexts. These factors are instead aggregated without distinction within the broad conceptual category of *moderating factors*.^35^

Considering uncertainty as a situated phenomenon, we used the *situativity theory* to explore situational characteristics and hypothesised that these would potentially determine participants' responses.^36^, ^37^ Situated cognition theory enabled us to identify individual characteristics (e.g. personal resources, emotional state and expertise), the nature of the clinical problem and the characteristics of the context, environment and patient in each interview. Our analyses focused on the reciprocal interactions between these factors and the behavioural responses implemented by participants while facing uncertain clinical situations.

### 2.2 Context

In France, medical students undergo 6 years of training. At the end of this period, they take the national residency exam, which qualifies them as residents specialising in either medicine or surgery specialties. Depending on the specialty, residents undergo 4 to 6 years of training, culminating in their certification as Doctor of Medicine. During their final year, residents are considered ‘junior doctors’ and are given a higher level of responsibility and autonomy, including working as independent consultants, referring back to their supervisor only if necessary.^38^

### 2.3 Population and sampling strategy

We hypothesised that sampling participants from different specialties would promote diversity in the situations encountered, thereby providing rich and nuanced experiences of clinical uncertainty. Several studies suggested that gender and clinical experience may be two important moderating factors in the experience of uncertainty.^39^, ^40^, ^41^, ^42^ Consequently, we used a maximum variation sampling strategy to ensure diversity in year of residency and gender among participants. In line with our conceptualisation of uncertainty as a situated experience, determined by contextual factors, we deliberately excluded residents from surgery and general medicine—the latter being mainly practiced in private settings—to focus our exploration on the hospital environment specific to medical specialties. The principal investigators sent an email invitation to all the institution's medical residents. The message specified that participation was voluntary and that no incentives or financial compensation would be provided. To avoid hierarchical influence and ensure unbiased participation, residents who had any member of the research team as a clinical supervisor were not approached for recruitment. Participants were selected among the volunteers to best reflect the diversity of the target population, according to the defined sampling framework. Interviews continued until data saturation was reached, which was defined as the absence of new themes emerging after two consecutive interviews.^43^

### 2.4 Conducting the interviews

The researchers jointly developed a semi‐structured guide for individual interviews, based on existing literature and their own experience, which was pre‐tested with a subset of residents not participating in the study. At the beginning of each interview, participants were asked to recall a situation in which they had felt particularly uncertain during a hospital placement. Subsequent questions aimed to explore how they reacted in depth, highlighting the resources they mobilised, the actions they took and how they questioned the situation to adjust their responses. All interviews were conducted by NB, recorded and transcribed verbatim.

### 2.5 Analyses

Data analysis was conducted iteratively and concurrently with data collection, gradually integrating the contributions of subsequent interviews into the development of codes. The first five verbatim transcripts were independently double coded by two investigators (NB and FR). Following a high level of agreement between coders and resolution of any discrepancies through discussion, NB coded the subsequent transcripts. Coding was conducted line by line with constant comparison, enabling new data to be continuously compared with established categories.

Our thematic analysis followed the six steps proposed by Braun and Clarke^30^: (1) familiarisation with the data; (2) generation of initial codes, produced deductively from the literature and inductively from the data; (3) searching for themes by grouping codes into coherent sets; (4) revising themes by comparing them with data extracts and discussing them within the research team; (5) defining and naming themes to clarify their scope and the relationships between them; and (6) producing the report by selecting representative, illustrative quotations. Notes and memos were kept to track the evolution of our understanding of the data during the analysis. The research team met regularly to discuss the codes, their organisation into categories and the interactions between themes, with the aim of strengthening the consistency of the analysis. We used NVivo (version 14.24.3) to organise, code and manage the data.

## 3 RESULTS

Between November 2023 and January 2025, we interviewed 14 residents at Rennes University Hospital (eight of whom were women) from nine different specialties: endocrinology, rehabilitation, nephrology, pulmonology, rheumatology, haematology, vascular medicine, psychiatry and gastroenterology. The participants' characteristics are described in Table 1. The interviews lasted between 32 and 55 minutes (mean = 45 minutes). Saturation was reached after 14 interviews, as no new themes were identified in the two final transcripts. The three overarching themes derived from Han's taxonomy—reducing uncertainty, protection and adaptation—adequately captured the range of behavioural responses described by the participants. Notably, the process of classifying uncertainty—that is, determining whether it could be reduced by gathering additional information—was mentioned by only one participant: ‘I think there are situations where, consciously, I tell myself I don't have the answer to this question, but I know someone else will.’ Participant 14, final year.

**TABLE 1:** Participants characteristics.

Characteristics	Participants *n* = 14
Age (years) (mean ± *SD*)	26.8 (±5.6)
Gender M/F	6/8
First year	5
Intermediate	3
Last year	6
First half/second half of residency	7/7

While participant 14 framed his uncertainty as ‘known unknowns’, other participants' behavioural responses were mainly shaped by situational factors, particularly the perceived urgency of the situation:

Today, I think it's mainly the degree of urgency that makes me seek out someone more experienced if I need an immediate answer to a question, because it has an immediate therapeutic impact on the patient. If I know that the problem can be postponed, I'll try to find the answer myself … Participant 12, final year.

Themes related to patient, participant, problem and environmental characteristics emerged as key situational factors. In the following sections, we present the response themes in relation to these salient situational factors, which participants identified as shaping or conditioning their behavioural responses.

### 3.1 Reducing uncertainty

The most frequently described responses aimed to gather additional information to reduce uncertainty. This led participants, particularly in situations involving time constraints and high risk for the patient, to seek supervisors' help. This approach was particularly salient in situations involving technical procedures:

Very recently, I had a patient with oesophageal cancer. I was unsure how to refeed him. On a Friday evening at 5 p.m., I attempted to insert a gastric tube using a small‐calibre fiberscope, but was unsuccessful. I hurt him and told him, ‘I'll arrange for your jejunostomy quickly’. But actually, I didn't even know if we could put him to sleep. My boss came by, she said, ‘There's nothing more we can do …’ so I checked with the surgeons, and he had the operation on Tuesday. Participant 9, Year 7.

Conversely, non‐acute situations prompted responses focused on independent problem‐solving rather than immediate risk management. Participants described planning further tests, reviewing the patient's file or trying a therapeutic test to reduce uncertainty. Delaying action to reassess or change approach was seen as particularly effective and was linked to experience level:

Looking back on the situation, I think I was a bit hasty … I was a young resident at the time! At the very beginning of my residency, when I encountered a problem, I wanted to solve it immediately … Whereas now, I go back over the patient's file, what we did and what has already happened. Overall, I feel that taking your time is quite important in many situations. Participant 7, Year 7.

### 3.2 Protection against the adverse effects of uncertainty

All participants described the cognitive and emotional effects of uncertainty in largely negative terms, citing anxiety, indecision and loss of confidence as key issues. These effects were linked both to the perception of high risk and to the fear of having their skills questioned, thereby reflecting participants' perceptions of self‐efficacy. Participants described responses aimed not at reducing the uncertainty, but at protecting themselves from it:

I currently have a patient for whom we have been unable to prove a relapse of lymphoma, despite numerous biopsies. The multidisciplinary team meeting approved starting a kind of trial treatment anyway. However, I don't think any of us would have made the decision to treat her alone. The multidisciplinary team meeting has a medico‐legal aspect, but it also provides us with individual relief because we make the decision collectively. Participant 12, final year.

Participant 12 noted that collegial discussion strengthened practitioners' ability to manage irreducible uncertainty, a defining feature of cases where diagnosis was difficult. In such situations, protective strategies—legitimising uncertainty, providing reassurance and sharing responsibility within the team—were key supports for residents. Some participants, particularly early in residency, expressed aversion to uncertainty and adopted avoidance strategies, often by transferring the issue to their supervisor:

Uncertainty makes me not want to go and see the patient … this fear of getting feedback, of having to negotiate, of being blamed … so I can wait for the rounds or even try to arrange for the senior consultant to go and see them instead. Participant 13, final year, talking about a situation he experienced at the start of his residency.

Conversely, some participants described how they sought to reassure themselves to cope with their anxiety, even sometimes seeking to occult their uncertainty:

I always try to find a solution to a problem, even if it's not necessarily the right strategy. Sometimes I get stuck in tunnel vision, telling myself, ‘This is what needs to be done’. That way, I can forget about everything else and reassure myself. It's not necessarily a very good thing … Participant 7, Year 7.

These protective responses were sometimes described as ‘compartmentalisation’, where participants distanced themselves from uncertainty by focusing on other tasks to mitigate its impact. This response typically arose in high‐stakes situations involving severe illness or patient suffering, often early in the day, allowing participants to ‘move on’ and continue caring for others while limiting emotional burden. More experienced participants also reported such emotions but sought instead to integrate uncertainty to make independent decisions. This heightened sense of responsibility and concern for patient outcomes sometimes paradoxically intensified their experience of uncertainty:

I saw a psychiatric patient in the ER who had been seen many times before and sent home each time. I saw him at the end of my shift, and he had very clear suicidal thoughts. I spoke to the head of the department, telling him that I thought the patient needed to be hospitalised. The next day, I looked at his file and saw that they had discharged him … In hindsight, I felt a bit bad about the situation and wondered if I had been clear enough and should have insisted more? Participant 11, final year.

### 3.3 Adapting to uncertainty

Participants described adapting to uncertainty as aiming to adjust their responses to take into account the undefined and dynamic nature of their situation:

The patient's sons were quite vehement at the beginning of the consultation. I wanted to find out if they understood why we had suggested there were no other treatment options, namely that this was a disease requiring treatment primarily focused on comfort. They understood that. However, contrary to what they had thought, not all treatments necessarily had an unfavourable risk–benefit ratio, particularly in terms of comfort. It might therefore be worthwhile to adopt a slightly more aggressive approach. Participant 14, final year.

Here, the participant describes seeking a decision‐making partnership with his patient and her family, comparing his objectives with theirs in order to adjust his decisions. This shared medical decision‐making strategy was used in situations where uncertainty arose from ethical dilemmas related to the level of care to be provided. It was closely associated with another adaptive, cognitive strategy consisting of participants adjusting their epistemic expectations:

At one point, I checked whether he needed resuscitation. Seeing that he didn't, I didn't call the resuscitators. I had spoken to the nursing team, who told me that his condition had deteriorated over 24–48 hours. His daughter left saying, ‘I won't see him again tomorrow’. The patient told me, ‘I'm in pain, I'm scared’. That's when I realised that something really did need to be done. So I refocused on concrete problems that I knew I would be able to solve in some way. Participant 2, Year 8.

Here, participant 2 describes how the evolving situation led her to modify her expectations about what could be known or predicted and to focus on achievable goals centred on the patient's well‐being rather than solely on curing the disease.

Some participants relied on pre‐established patterns, such as algorithms, to reorganise their perception of the problem and give it logical coherence:

To manage these situations, I have my diagnostic and decision‐making tree in my head. Participant 1, Year 10.

Conversely, when faced with unfamiliar situations, other participants developed a specific action plan as the situation evolved:

I don't think he met the criteria for hospitalisation. I thought we could admit him the following week—in any case, there were no beds available over the weekend. I wasn't going to rush him into another department on a Friday. I knew they were going to have a rough weekend emotionally and in terms of stress, but clinically, he was not in any danger for the next two days. Also, it was complicated because his metastatic status was unclear, so decisions would have to be made and a lot of discussion would be necessary. Participant 9, Year 10.

Participant 9 illustrates how she integrates contextual information, resource availability and situation severity to prioritise issues, forming a structured, dynamic problem representation. These adaptive responses required distancing from the situation's immediacy and were closely linked to experience level.

Stress and fatigue were described as major obstacles to taking an organised and structured approach to the problem:

It was the end of my shift and I hadn't slept. I couldn't find a pulse or take his blood pressure. In short, it took ages to take his vital signs. I didn't think to take his pulse manually because… I was too tired. I didn't stop to think because I was far too stressed by the situation. Participant 3, final year.

**FIGURE 1:**
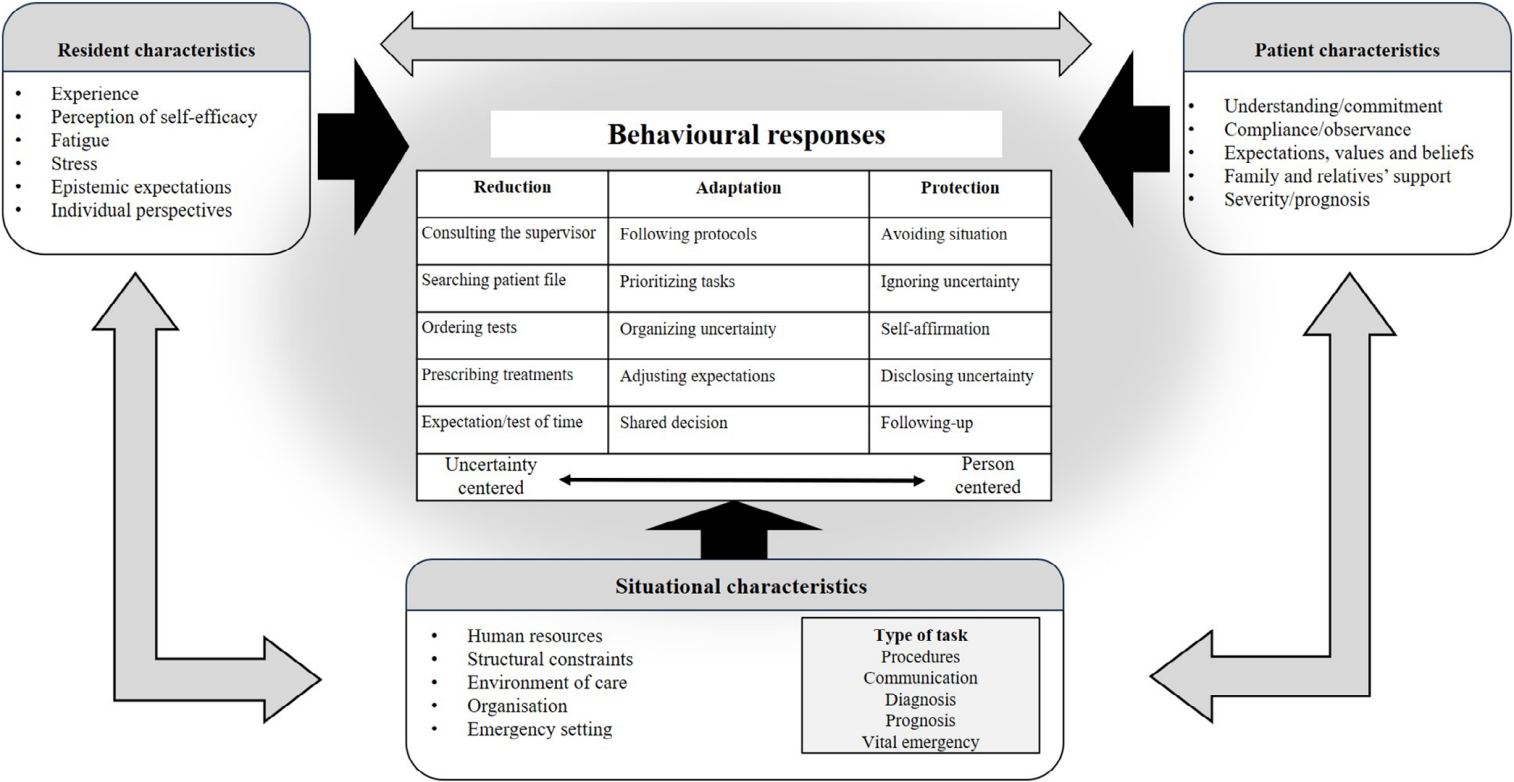
Conceptual map illustrating the relationships between residents' behavioural responses and situational characteristics. Figure 1 shows the main themes relating to the behavioural responses of the participants and the situational characteristics that influence these responses. Although this model does not present any directional or hierarchical relationships between the themes, as these were specific to each situation, it does highlight the situational characteristics that participants described as influencing their decision‐making processes. These characteristics were therefore described as salient cues within their situations. [Color figure can be viewed at wileyonlinelibrary.com]

Figure 1 illustrates how the experience of clinical uncertainty emerges from the reciprocal interactions between key individual characteristics, patient‐related features and situational characteristics including both the environment and the task at hand. These interacting factors shape residents' responses to uncertainty, which range from uncertainty‐reducing strategies to adaptive, patient‐centred approaches, forming a continuum from *uncertainty‐centred* to *person‐centred* responses. Although the model does not assign any fixed directional or hierarchical relationships among these themes—because their relevance varied across situations—it highlights the situational characteristics that participants identified as shaping their decision making. These characteristics therefore functioned as salient cues within their clinical encounters.

**FIGURE 2:**
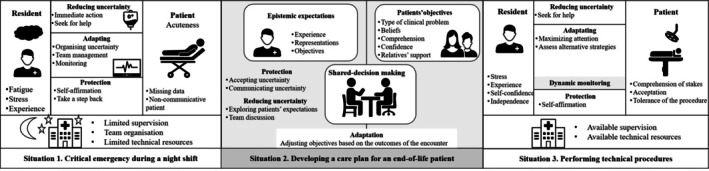
Illustration of the relationships between the main themes through three situations discussed by participants. These three clinical vignettes, taken from the verbatims of three participants, highlight the link between behavioural responses and the characteristics of the situations. These situations, described as frequent, particularly significant and high‐stakes, were highly uncertain and may thus be considered as ‘integrative problem situations’.

Figure 2 further contextualised the relationships between the main themes through three clinical situations discussed by participants. These three vignettes, drawn directly from participants' verbatim, highlight the dynamic link between residents' behavioural responses and the specific characteristics of the situations they encountered. Each vignette reflects a scenario described as frequent, particularly significant and high stakes. These highly uncertain encounters may therefore be considered ‘integrative problem situations,’ where multiple factors converge to shape the residents' experience and management of uncertainty.

### 3.4 Evolution of responses to uncertainty during residency: Towards a situated and systemic approach

Throughout their residency, the most experienced participants gradually became more comfortable with uncertainty. This comfort was mainly due to their accumulated experience, which enabled them to identify the key characteristics of each situation and triggered responses that were perceived as more reliable and appropriate. Above all, accepting uncertainty and recognising its inherent, unavoidable nature in certain health care situations led them to adopt a systemic approach. From this perspective, they described mobilising different strategies simultaneously, with the aim of acting on the source of uncertainty by enriching the available data on which to base decisions, protecting themselves from its effects and adjusting their objectives according to the evolution of the situation.

**FIGURE 3:**
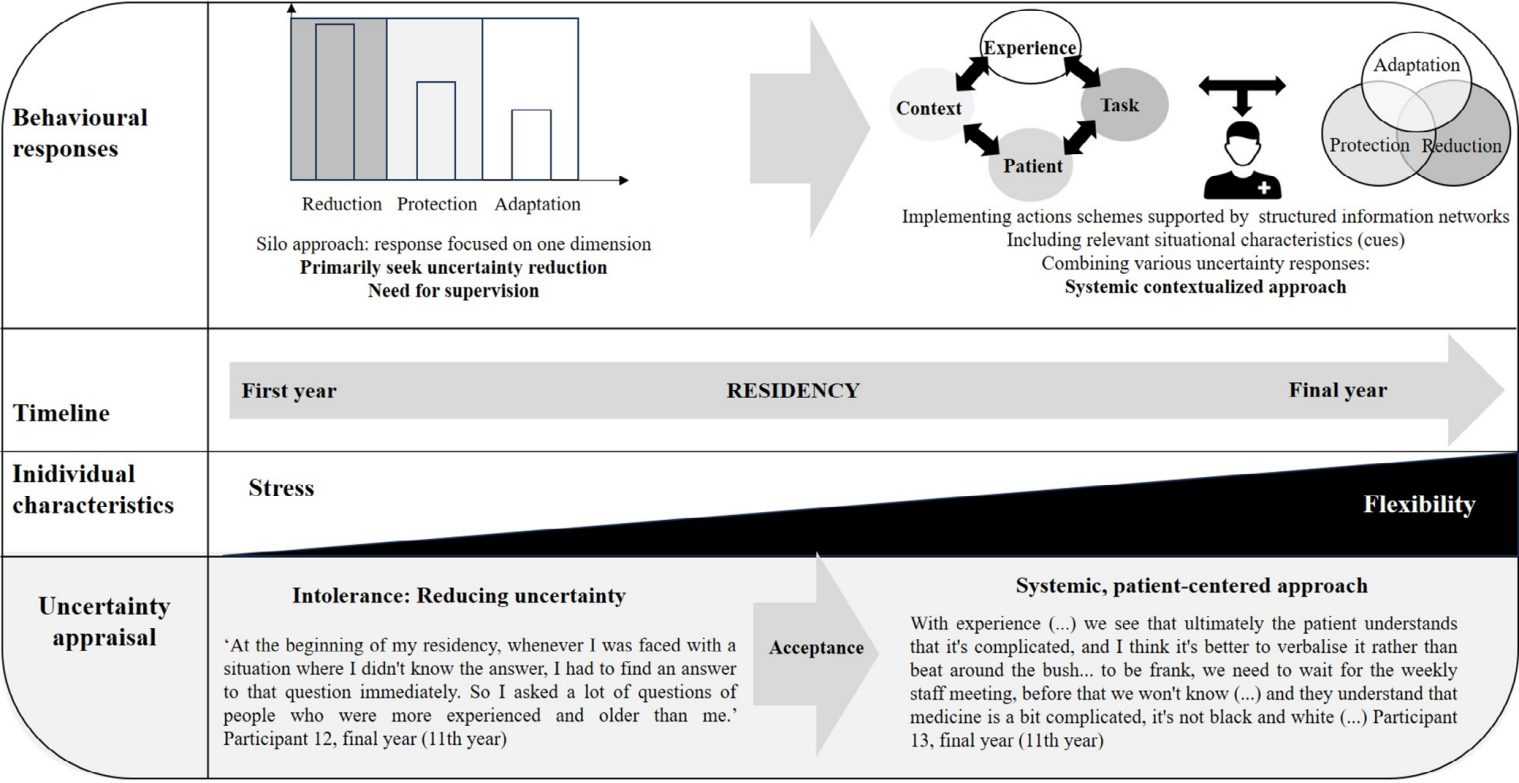
Evolution of residents' behavioural responses during residency in relation to their appraisal of uncertainty. [Color figure can be viewed at wileyonlinelibrary.com]

Transforming their relationship with uncertainty—shifting from resistance to acceptance—enabled participants to share it with patients and integrate it into their decision‐making processes. Searching for agreement between doctors' and patients' objectives then helped reduce uncertainty while increasing clinicians' comfort. Figure 3 summarises the evolution of the main themes throughout the course in relation to the participants' relationship with uncertainty. Thus, the development of comfort with uncertainty, along with experience, was based on a combination of complementary strategies aimed at providing an adequate response to the various issues identified. This evolution fostered a sense of competence and comfort when navigating complex situations, as participant 1 illustrates:

When I arrived, there were too many people involved with this agitated patient who was shouting, wanting to leave, and refusing all our suggestions. Things then started to get a little agitated around him. I think some of the staff members were shouting too, so I went to the medical office with a nurse, a quiet place where she could explain the situation to me in detail. When she had explained it to me, I asked her, ‘What do you think we should do?’ She was a fairly experienced nurse whom I trusted. I sought her advice before returning to face the situation and explain to the patient what I was going to do. Participant 1, final year.

## 4 DISCUSSION

This study sheds light on how residents respond to situations of uncertainty in their clinical practice. The diversity of our participants' specialities and experiences led to discussions about a wide range of work situations, emphasising the contextual nature of uncertainty management in clinical settings. Relying on the situativity theory to explore residents' stories resulted in a view of clinical uncertainty as a subjective experience resulting from dynamic interactions between the environment, the problem at hand and residents' own characteristics. Among them, experience, personal values, self‐confidence and trust in the system and their supporting environment were depicted as essential characteristics shaping their situational appraisal, according to previous work highlighting the feelings of insecurity of junior doctors when confronted with uncertainty.^44^

In line with the taxonomy of sources (probability, ambiguity, complexity) developed by Han,^1^, ^13^ Helou et al. proposed a conceptual model of decision making under uncertainty centred on an initial stage of diagnosing whether the stimulus is reducible or not.^23^ Such a process would be grounded in reflection, allowing residents to distinguish between Renée Fox's three types of uncertainty: type 1 (lack of personal knowledge), type 2 (the inherent limitations of medical knowledge) and type 3 (difficulty in recognising or articulating one's own uncertainty).^24^ In our study, the irreducible aspect of uncertainty ‐ reflecting its ontological nature‐ emerged as a situational factor shaping participants' responses. Participants tended to adopt protective strategies, such as shared decision making, to mitigate the adverse effects of uncertainty. However, no participant explicitly described the ‘categorisation of uncertainty’ as an initial step in their decision‐making process. Several hypotheses may explain this discrepancy.

First, Helou's model is based on analyses of decision‐making models developed in various disciplines (e.g. psychology, economics and management), with little representation from health care professionals. Therefore, its transferability to the medical field remains uncertain and even more so to medical trainees.

Second, acute immersion in high‐stakes, time‐constrained situations is likely to limit the mobilisation of metacognitive reflection. Prior research shows that reflection is only sparingly used in routine decision‐making tasks.^26^, ^27^, ^28^ Although uncertainty is often conceptualised as a hallmark of analytic, system 2 reasoning, evidence suggests that deliberately triggering this process is time consuming and may not be feasible under real clinical conditions.^45^

In our study, participants primarily relied on an operational decision‐making approach focused on effective action. Their choice of responses to uncertainty was guided by an implicit assessment of their affordances and effectivities—the internal and external resources available to act appropriately.^36^ This assessment was shaped by two key indicators, which were time constraints and clinical experience. This time factor was particularly relevant in situations of emergencies or involving technical procedures. In these contexts, residents drew on prior knowledge to make rapid, ‘automatic’ decisions, precluding deliberate analysis of the root cause of uncertainty. While this highlights a difference between residents and models developed for more experienced practitioners, whether it compromises decision quality remains debated. Current evidence suggests that fostering system 2 reasoning does not necessarily improve clinicians' ability to navigate complex or uncertain tasks, underscoring how reasoning in practice strongly relies on intuitive, system 1 reasoning, shaped by contextual demands.^46^, ^47^, ^48^ To our knowledge, no study has specifically examined how time constraints influence reasoning in situations of genuine clinical uncertainty.

Yet this strong influence of time constraints on residents' response strategies also highlights the central role of experience in shaping their decisions. Our results suggest that less experienced trainees are particularly affected by uncertainty, as they struggle to develop a reliable plan of action under pressure. Experience therefore appears crucial in enabling residents to organise knowledge efficiently and make rapid decisions, especially in acute situations marked by stress, severity, or limited time.

On the other hand, the most experienced participants demonstrated adaptation‐oriented responses, monitoring the impact of their decisions on the situation's overall evolution. They used various contextual, organisational, relational and biomedical indicators to continuously adjust their representations and objectives. This approach builds on the theory of cues, used in previous studies exploring decision making under uncertainty by adopting a systemic and ecological perspective in line with situativity theory.^20^, ^21^, ^49^ In this theory, the characteristics of the situation are integrated to inform the action plan accounting for the dynamics and instability of clinical contexts.

From this perspective, integrating the patient as a decision‐making partner also appeared to be an indicator of experience. Initially perceived as a constraint and an additional source of uncertainty, interactions with patients gradually became an essential resource for the participants in constructing a shared decision. From a situated perspective, patients can be understood as contextual determinants in their own right; their values, preferences and clinical narratives shape uncertainty and determine the range of possible decisions. This is consistent with research on shared medical decision making, which emphasises that uncertainty is not merely reduced, but rather redistributed and co‐constructed within the physician–patient interaction.^50^, ^51^

Over the course of their training, residents developed not only a broader repertoire of responses but also the ability to combine them. This flexibility enabled them to address the sources of uncertainty simultaneously, protect themselves from it and integrate it into their reasoning. In this sense, our results align with Han's view that flexibility is a key virtue for responding to uncertainty.^13^, ^52^ Several studies have employed quantitative approaches to measure the relation between the level of experience and uncertainty tolerance in medical trainees, using a variety of instruments.^5^, ^53^, ^54^, ^55^ Although these studies yield inconsistent results regarding whether tolerance improves across their curriculum, the evolution in residents' decision making under uncertainty highlighted by our study offers important insight into the way experience underlies the improvements observed in some of these research. In other words, becoming comfortable with uncertainty requires an attitude of acceptance and integration, sustained by clinical experience and the effective combination of situated strategies.

### 4.1 Limitations

This study has several limitations. Its single‐centre design may have restricted the richness of the data, although the diversity of the participants' backgrounds and placements supported varied and lively discussions. Notably, all participants had completed at least one rotation in another hospital, thereby gaining exposure to different professional environments. Although thematic saturation was achieved, the sample size did not allow for comparisons of strategy frequency across training stages, a question that would require a larger‐scale quantitative design. Finally, irreducible uncertainty—reflecting ontological uncertainty that cannot be resolved by reference to normative standards—precluded any judgement of the quality or relevance of the responses described by participants. Nevertheless, an evaluative approach would be valuable for two reasons. First, it could help to determine whether residents' perceived comfort is a reliable indicator of their ability to manage clinical uncertainty. Second, it could assess the effectiveness of their response processes and whether the absence of an explicit uncertainty‐classification step, as described in other models, limits performance. From this perspective, future work could build on the principle of decisional concordance, as operationalised into learning by concordance, to evaluate the appropriateness of responses mobilised under uncertainty.^56^

### 4.2 Implications for medical education

Our findings highlight that uncertainty management is inherently contextual and, akin to clinical reasoning, cannot be meaningfully examined apart from the situation in which it occurs. Explicit supervision, transparent decision making and regular debriefing are essential to help residents develop comfort with uncertainty and prevent the harms of facing it unsupported. The problem situations identified could inform the design of training and assessment scenarios focused on uncertainty, in line with the concept of EPAs.^57^ They could also guide simulation activities, serving as both learning and iterative assessment tools within a programmatic approach that recognises uncertainty management as a core medical competency.

### 4.3 Implications for research

Given its cross‐sectional design, our study cannot describe the individual developmental trajectories of responses to or tolerance of uncertainty. These trajectories are likely to vary widely across individuals and are influenced by personal characteristics and personal representations of uncertainty. Longitudinal studies are needed to model these trajectories and analyse the evolving dynamics of responses to uncertainty throughout training.

## 5 CONCLUSIONS

Our study shows that residents' comfort with uncertainty develops progressively through complementary, context‐sensitive responses. With experience, their relationship to uncertainty evolves towards acceptance and integration, supported by flexible strategies adapted to diverse situations. Uncertainty thus appears as a situated experience shaped by dynamic interactions between practitioners and their environment, echoing Dewey's pragmatist view of practice as embedded within and shaped by the environing world.^58^ Recognising this situated nature moves us beyond a purely cognitive view, framing uncertainty as a core competency cultivated through experiential learning in meaningful clinical encounters.

## AUTHOR CONTRIBUTIONS

Nicolas Belhomme and François Robin made substantial contributions to the conception and design of the study, recruited participants and carried out data collection and analysis. They jointly drafted the manuscript and provided critical revisions. Alain Lescoat, Pierre Pottier, Yoann Launey, Emmanuel Triby and Thierry Pelaccia contributed significantly to the study's conception and design, offered substantial input to the data analysis and critically revised the manuscript. All authors gave final approval to the submitted paper and are accountable for all aspects of the work.

## CONFLICT OF INTEREST STATEMENT

None declared.

## ETHICS STATEMENT

This study was approved by the Ethics Committee of Rennes University Hospital (opinion no. 21.178).

## DECLARATION OF GENERATIVE AI AND AI‐ASSISTED TECHNOLOGIES IN THE WRITING PROCESS

DeepLPro software was used solely for grammar and spelling editing to improve the readability and language. After using this tool/service, the authors reviewed and edited the content as needed and take full responsibility for the content of the published article. No AI was used for any other step; in particular, content generation or interpretation were not performed or assisted by AI.

## Data Availability

The data that support the findings of this study are available on request from the corresponding author. The data are not publicly available due to privacy or ethical restrictions.
